# The loading direction dramatically affects the mechanical properties of the mouse tibia

**DOI:** 10.3389/fbioe.2024.1335955

**Published:** 2024-02-06

**Authors:** Saira Mary Farage-O’Reilly, Vee San Cheong, Edmund Pickering, Peter Pivonka, Ilaria Bellantuono, Visakan Kadirkamanathan, Enrico Dall’Ara

**Affiliations:** ^1^ Insigneo Institute for in silico Medicine, University of Sheffield, Sheffield, United Kingdom; ^2^ Healthy Lifespan Institute, University of Sheffield, Sheffield, United Kingdom; ^3^ Division of Clinical Medicine, University of Sheffield, Sheffield, United Kingdom; ^4^ Future Health Technologies Programme, Singapore-ETH Centre, Singapore, Singapore; ^5^ Department of Automatic Control and Systems Engineering, University of Sheffield, Sheffield, United Kingdom; ^6^ School of Mechanical, Medical and Process Engineering, Queensland University of Technology, Brisbane, QLD, Australia; ^7^ Centre for Biomedical Technologies, Queensland University of Technology, Brisbane, QLD, Australia

**Keywords:** micro-FE, micro-CT, bone strength, loading direction, mechanical loading, ovariectomy, mouse tibia, bone deformation

## Abstract

**Introduction:** The *in vivo* tibial loading mouse model has been extensively used to evaluate bone adaptation in the tibia after mechanical loading treatment. However, there is a prevailing assumption that the load is applied axially to the tibia. The aim of this *in silico* study was to evaluate how much the apparent mechanical properties of the mouse tibia are affected by the loading direction, by using a validated micro-finite element (micro-FE) model of mice which have been ovariectomized and exposed to external mechanical loading over a two-week period.

**Methods:** Longitudinal micro-computed tomography (micro-CT) images were taken of the tibiae of eleven ovariectomized mice at ages 18 and 20 weeks. Six of the mice underwent a mechanical loading treatment at age 19 weeks. Micro-FE models were generated, based on the segmented micro-CT images. Three models using unitary loads were linearly combined to simulate a range of loading directions, generated as a function of the angle from the inferior-superior axis (θ, 0°–30° range, 5° steps) and the angle from the anterior-posterior axis (ϕ, 0°: anterior axis, positive anticlockwise, 0°–355° range, 5° steps). The minimum principal strain was calculated and used to estimate the failure load, by linearly scaling the strain until 10% of the nodes reached the critical strain level of −14,420 με. The apparent bone stiffness was calculated as the ratio between the axial applied force and the average displacement along the longitudinal direction, for the loaded nodes.

**Results:** The results demonstrated a high sensitivity of the mouse tibia to the loading direction across all groups and time points. Higher failure loads were found for several loading directions (*θ* = 10°, *ϕ* 205°–210°) than for the nominal axial case (*θ* = 0°, *ϕ* = 0°), highlighting adaptation of the bone for loading directions far from the nominal axial one.

**Conclusion:** These results suggest that in studies which use mouse tibia, the loading direction can significantly impact the failure load. Thus, the magnitude and direction of the applied load should be well controlled during the experiments.

## 1 Introduction

Bone is a dynamic tissue that adapts over time due to biomechanical and biochemical stimuli (Martin et al., 1998; Frost, 2003; Bonewald, 2011). It is the balance between these adaptations which keeps bone healthy, through bone cells which continuously maintain and remodel the bone tissue. However, musculoskeletal diseases, such as osteoporosis, can disrupt this balance. Osteoporosis affects bone remodeling, reducing the bone mineral density (BMD) by reducing the number or thickness of trabeculae, reducing the cortical thickness, increasing the cortical porosity and/or decreasing the local mineralization and consequently the tissue mineral density (TMD). These changes deteriorate the mechanical properties, leading to a decrease in bone strength, and hence, an increase in fracture risk (Birkhold et al., 2014; Razi et al., 2015a; Roberts et al., 2019).

Various treatments exist for osteoporosis, including antiresorptive treatments (targeting osteoclasts, i.e., bone resorbing cells) or bone anabolic treatments (targeting both osteoclasts and osteoblasts, i.e., bone forming cells). However, these treatments have side effects, for example, long term use of bisphosphonates (antiresorptive drugs) is associated with atypical femoral fractures (Shane et al., 2014). Anabolic treatments such as Parathyroid Hormone (PTH) are associated with poor cost effectiveness (Yeam et al., 2018). Therefore, new and improved treatments are needed. New treatments must be tested in animals preclinically before being assessed in clinical trials, with the mouse model being the most commonly used animal model in drug development (Gould et al., 2015). Osteoporosis treatments are usually tested in adult mice after ovariectomy, a model of estrogen deficiency that induces accelerated bone resorption and a phenotype similar to that observed in postmenopausal osteoporotic patients (Bouxsein et al., 2005; Roberts et al., 2019). Mechanical loading affects bone adaptation responses (Frost, 2003). Low levels of mechanical loading (including complete unloading) can cause bone resorption, whilst high levels of mechanical loading can stimulate bone formation, hence, increasing bone density and bone strength (Frost, 2003). As a result, mechanical loading in the form of exercise has been suggested as an anabolic treatment against osteoporotic fractures (Martelli et al., 2020; Du et al., 2021; O’Rourke et al., 2021). Additionally, mouse models have been used to study the combined effects of mechanical loading and pharmacological treatments on bone remodeling and mechanical properties (Levchuk et al., 2014; Scheuren et al., 2020).

Several rodent models are available to evaluate the effect of mechanical loading on the bone in a controlled way (Nepal et al., 2023). Among them, the *in vivo* tibial loading model has been extensively used to evaluate bone adaptation in the tibia after the application of external mechanical loads through the knee and the ankle joints, aiming at loading the tibia predominantly along its axial direction. This model has been used to investigate bone adaptation in various ways, such as: in disuse models (Moustafa et al., 2012); under different peak loads, by mimicking impact exercises (De Souza et al., 2005; Sugiyama et al., 2012; Main et al., 2020; Miller et al., 2021); varying loading type (static vs. static and dynamic) (Sugiyama et al., 2010); and varying the load frequency (Holguin et al., 2013; Yang et al., 2017). Investigations into the effect of mechanical loading on bone adaptation in aged mice (Lynch et al., 2011; Birkhold et al., 2014; Razi et al., 2015a, 2015b), ovariectomized mice (Roberts et al., 2019, 2020, 2023) and healthy mice (Melville et al., 2014) have shown an increase in bone formation. Furthermore, some studies have looked at increasing the bone anabolic effect of mechanical loading over time on the mouse tibia through the administration of drug treatments, specifically PTH (Sugiyama et al., 2008; Meakin et al., 2017; Cheong et al., 2020a; Roberts et al., 2020; Rooney et al., 2023), working towards the optimization of combined biomechanical and pharmacological osteoporotic treatments.

Although the *in vivo* tibial loading model is widely used, there is a prevailing assumption that the load is applied axially to the tibia. However, considering the transmission of the applied load through the knee and ankle joints, compounded by the potential for the leg to be repositioned within the loading device during longitudinal studies, uncertainty arises over the true loading direction and the effect that this may have on bone adaptation. Digital image correlation measurements have highlighted the sensitivity of the mouse tibia superficial strain as a function of the loading conditions (Carriero et al., 2014). Moreover, a previous study that used digital volume correlation (DVC) showed that repositioning of the bone when using the *in vivo* tibial loading model affected the internal strain distribution across the tibia, inducing areas of higher strain, particularly at the distal end of the bone (Giorgi and Dall’Ara, 2018).

The gold standard for evaluating the effect of mechanical loading and combined treatments on bone densitometric and morphometric properties is using *ex vivo* or *in vivo* micro-computed tomography (micro-CT) (Bouxsein et al., 2010). Recently, micro-CT based micro-finite element (micro-FE) models have been developed to non-invasively evaluate the mechanical properties of bone. Micro-FE models not only have the potential to dramatically reduce and partially replace the use of mice in skeletal research (Viceconti and Dall’Ara, 2019), but also to evaluate the sensitivity of different parameters on the biomechanical properties of the bone; something that could not be achieved with experiments. Nevertheless, before their application in preclinical studies, the models should be validated against biomechanical experiments. Recent validation studies showed that hexahedral homogeneous micro-FE models can accurately predict local displacements (*R* ^2^ > 0.82, against displacements measured with DVC), apparent stiffness (errors of 14% ± 11%), and failure load (errors of 9% ± 9%) (Oliviero et al., 2018). Following that study, an optimization of the failure criterion in the mouse tibia was conducted. Hexahedral homogeneous micro-FE models were found to accurately predict normalized stiffness (*R* ^2^ = 0.80, error of 14% ± 8%) and normalized failure load (*R* ^2^ = 0.81, errors of 9% ± 6%) (Oliviero et al., 2021a). Different material properties (homogenous and heterogeneous) and different mesh types (hexahedral and tetrahedral) were also investigated. The micro-FE models with homogeneous material properties and hexahedral meshes were found to be the best predictors of the tibial mechanical properties (Oliviero et al., 2021b). Additionally, strain gauges have been used to compare local experimental strain measurements to the predictions of micro-FE models within the same locations (Stadelmann et al., 2009; Patel et al., 2014; Yang et al., 2014; Razi et al., 2015b). The accuracy of the micro-FE models for the estimation of the local strains varied (0.40 < *R* ^2^ < 0.99, errors between 5% and 20%). It should be considered that several factors contribute to the comparison between experimental and computational strain assessments, including the number and location of strain gauges, differences in the ages of the mice used, and variations in micro-FE input parameters.

Micro-FE models combined with strain gauges placed on the lateral, posterior and antero-medial surfaces of the bone have been used to inversely identify the point of application of the external load. It was found that the load location varied among mice, and the strains in the tibia were highly sensitive to the load location (Pickering et al., 2021). Micro-FE models have also been used to investigate the loading conditions when predicting bone adaptation during application of the *in vivo* tibial loading model, highlighting the importance of the load direction (i.e., the magnitude of the three Cartesian components of the load), through the inclusion of both the external axial load and the daily physiological axial and transverse loads (Cheong et al., 2021a). Nevertheless, the extent to which the mechanical properties of the mouse tibia are affected by the loading direction associated with the tibial loading model is still unknown.

The aim of this study was to evaluate how much the apparent mechanical properties of the mouse tibia are affected by the loading direction, by using a validated micro-FE model of mice which have been ovariectomized and exposed to external mechanical loading over a two-week period.

## 2 Materials and methods

### 2.1 Experimental *in vivo* data

**FIGURE 1:**
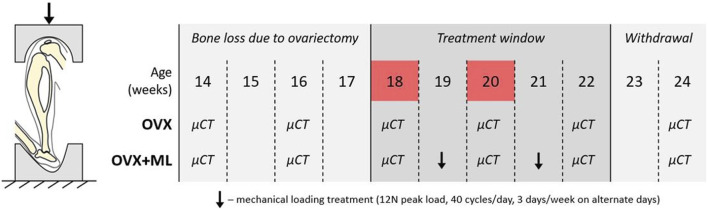
Left: Schematic of the *in vivo* tibial loading model. Right: Overview of the data collection timeline in C57BL/6 mice, acquired from a previous study (Roberts et al., 2020). OVX: ovariectomy, OVX + ML: ovariectomy and mechanical loading. Ovariectomy was performed at age 14 weeks, micro-CT images were taken every 2 weeks throughout the duration of the study, treatment window commenced at week 18 and was withdrawn by week 23. Images from weeks 18 and 20 were used in this study (highlighted in red).

The experimental data used in this study were acquired from a previous study by Roberts et al. (2020), wherein it was determined that a sample size of six mice per group was sufficient to attain 80% statistical power, considering morphometric parameters such as trabecular bone volume fraction and cortical thickness. Eleven female C57BL/6 mice were subjected to ovariectomy (OVX) at age 14 weeks (Figure 1). *In vivo* micro-CT images were taken of the right tibiae of all mice every other week, from week 14 to 24 (VivaCT80, Scanco Medical Brütisellen, Switzerland). The scanning protocol used (55 kVp, 145 μA, 10.4 μm isotropic voxel size, 32 mm field of view, 100 ms integration time and 1,500/750 samples/projections) allowed for minimal effects of radiation on the tibia whilst still allowing for scanning of the whole bone at high resolution (Oliviero et al., 2017, 2019). The images were reconstructed using a third-order polynomial beam hardening correction algorithm based on a 1,200 mgHA/cm^3^ wedge phantom, which was provided by the manufacturer.

In this study the micro-CT images acquired both for the ovariectomized mice (OVX group, N = 5) and for mice ovariectomized and subsequentially subjected to external mechanical loading (OVX + ML group, N = 6) were considered. Only images acquired at weeks 18 and 20 were used. Briefly, mice in the OVX + ML group underwent external mechanical loading treatment at weeks 19 and 21 (Figure 1), using the *in vivo* tibial loading model. Each right tibia was fixed in between two soft caps and mechanically loaded using a 12 N peak load (2 N static preload superimposed with a 10 N high-strain dynamic load at a rate of 160,000 N/s (maximal nominal speed of the machine), 40 cycles/day, 3 days/week on alternate days; ElectroForce BioDynamics 5100, TA instruments, USA). The applied nominal load was assumed to be along the axial direction of the tibia. This procedure has been shown to induce cortical and trabecular lamellar bone adaptation without inducing micro-damage (De Souza et al., 2005). All the experimental procedures complied with the UK Animals (Scientific Procedures) Act 1986 and were approved by the local Research Ethics Committee of the University of Sheffield.

### 2.2 Image processing and micro-FE models

**FIGURE 2:**
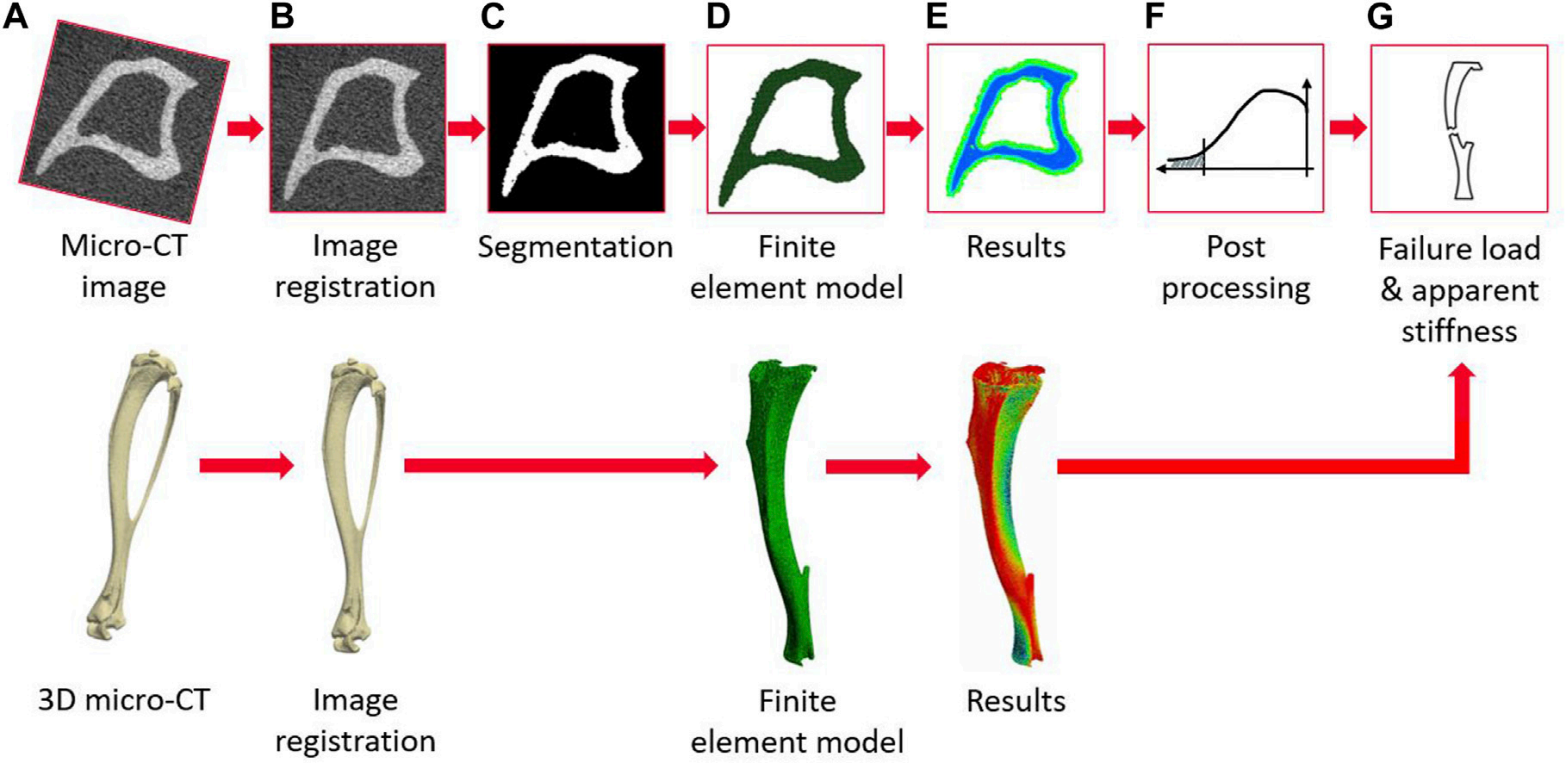
Flowchart illustrating the main steps of the pipeline to create the micro-FE models from the micro-CT images and to evaluate the apparent mechanical properties (from **(A–G)**) micro-CT acquisition, image registration, image segmentation (binarization), creation of the micro-FE models, results generation, post-processing of the local strains, calculation of failure load and apparent stiffness.

The main steps of the image processing, creation of the micro-FE models, and post-processing to calculate the apparent mechanical properties of each tibia are reported in Figure 2. The modeling pipeline has been previously validated for predictions of apparent structural properties using compressive tests (Oliviero et al., 2021b) and of local displacements using DVC (Oliviero et al., 2018).

To align the micro-CT images across the time points, the fibula was first virtually removed from all the images (MATLAB, 2018A, The MathWorks Inc., Natick MA, USA). One reference tibia was roughly aligned such that the axis of the bone was aligned to the *z*-axis of the image, and the sagittal plane bisected the midpoint of the line joining the centers of the articular surfaces of the medial and lateral condyles (Lu et al., 2016). This orientation is referred to as the “nominal axial” orientation within this study. The images were then rigidly registered to the reference image taken at week 14 (Amira 6.3.0, Thermo Fisher Scientific, France), as detailed in Lu et al. (2016, 2017). After alignment, the images were cropped from the slice below the proximal growth plate towards the distal end of the tibia, resulting in 80% of the total tibia length (Cheong et al., 2020b; Cheong et al., 2021b). This procedure was associated with reproducibility errors in estimating local bone mineral content (BMC) lower than 3.5% (Oliviero et al., 2022).

**FIGURE 3:**
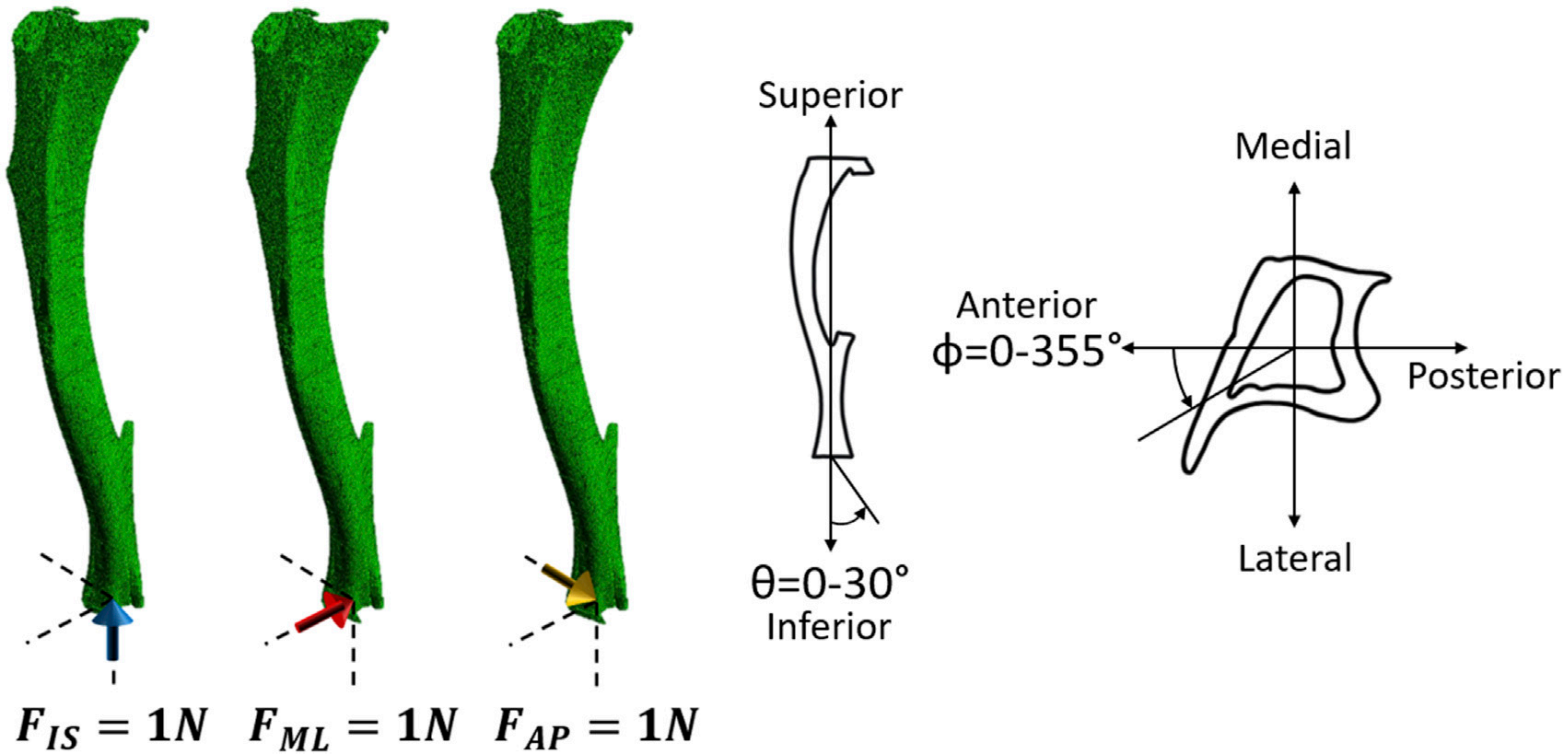
Left: Three independent unitary load cases (1 N in the inferior-superior direction (F_IS_), 1 N in the medio-lateral direction (F_ML_), and 1 N in the anterior-posterior direction (F_AP_) for each mouse at each time point) were used to calculate the failure load along the different loading directions. Right: Schematic representation of the loading directions as a function of the two angles *θ* and *ϕ*. *θ* describes the angle from the inferior-superior axis and ranges from 0° to 30° in steps of 5°. *ϕ* describes the angle from the anterior-posterior axis, going anticlockwise, and ranges from 0° to 355° in steps of 5°.

The images were segmented by applying a single-level threshold, defined as the midpoint between the background and bone peaks of the grey value histogram of the images (Oliviero et al., 2018; Cheong et al., 2021b). All segmented images were converted into micro-FE models by converting each bone voxel into a finite element (linear 8-node hexahedral elements). Larger elements were not used as they would not enable a proper description of the geometry of the trabecular bone in the proximal portion of the tibia (average trabecular thickness of approximately 45 μm (Roberts et al., 2020)). Tetrahedral elements were not found to improve the prediction of the bone mechanical properties (Oliviero et al., 2021b). Each model contained approximately 10 million nodes and 9 million elements. Isotropic, homogeneous, linear elastic material properties were used (E = 14.8 GPa, *v* = 0.3), which is in line with previous validation studies which showed good agreement between experimental measurements (Oliviero et al., 2018; Oliviero et al., 2021a; Oliviero et al., 2021b). In fact, heterogeneous material properties based on the local or average values of TMD calculated from the micro-CT images did not improve the predictive ability of the micro-FE models (Oliviero et al., 2021b). The boundary conditions were set to simulate the *in vivo* tibial loading model: the nodes in the proximal end were fully constrained, and the nodes in the distal end were connected via kinematic coupling to a control node which was located at the centroid of the distal surface with a small offset in the superior direction. This was done to avoid over-constraining the tibia (Cheong et al., 2020a). Three independent unitary load cases (Figure 3) were applied along the inferior-superior, medio-lateral or anterior-posterior directions for each mouse at each time point. The minimum principal strain was calculated at the nodes. All input files for the models were generated in MATLAB. The models were solved in Abaqus 2018 (Dassault Systèmes Simulia, RI, USA) using the University of Sheffield High Performance Computing Clusters (ShARC).

### 2.3 Post-processing

Due to the linear nature of the models, scaling and superposition of the effects were used to combine the results from the unitary load models during post-processing, to calculate the minimum principal strain for a resultant of 1 N with different combinations of loading directions (Eqs 1–4). Calculations were performed as a function of the angle from the inferior-superior axis (*θ*, 0°–30° range, 5° steps) and the angle from the anterior-posterior axis (*ϕ*, 0°: anterior axis, positive anticlockwise, 0°–355° range, 5° steps) (Figure 3).

$$\varepsilon_{\min}^{\theta ,\phi }=F^{IS}\left[\varepsilon_{\min}^{IS}\right]+F^{ML}\left[\varepsilon_{\min}^{ML}\right]+F^{AP}\left[\varepsilon_{\min}^{AP}\right]$$ (1)

where, $\varepsilon_{\min}^{\theta ,\phi }$ is the minimum principal strain value calculated in each node for the loading direction defined by the angles *θ* and *ϕ*, $\varepsilon_{\min}^{IS},\varepsilon_{\min}^{ML}{,and\;\varepsilon }_{\min}^{AP}$ are the minimum principal strain values calculated in each node for each of the three unitary cases, and $F^{IS}$ , $F^{ML}$ , and $F^{AP}$ are scaling coefficients, such that:

$$F^{IS}=\cos\theta$$ (2)

$$F^{ML}=\sin\theta \sin\phi$$ (3)

$$F^{AP}=\sin\theta \cos\phi$$ (4)

The bone strength (failure load; FL_FE, N) for each loading direction (Figures 2F, G) was estimated using a previously validated modeling pipeline (Oliviero et al., 2021a). The bone was assumed to fail when 10% of the nodes of the model reached a critical third principal strain value equal to −14,420 με Eqs. 5, 6 (Oliviero et al., 2021a). Therefore, the tenth percentile value of the minimum principal strain was calculated and used to rescale the applied unitary load in order to calculate the failure load.

Let $X=\left\{x_{1},x_{2},\ldots ,x_{n}\right\}$ be the ordered set of minimum principal strain values, where n is the number of nodes in the micro-FE model, then

$$FL\_FE=\frac{-14420}{x_{k}\times 10^{6}}\times F$$ (5)

where, F is the force applied to the micro-FE model (in this case 1N) and

k=⌈0.1n⌉ (6)

i.e., k is the smallest integer greater than or equal to 10% of n .

For each loading direction, the normalized failure load (NFL_FE, N) was calculated as the FL_FE calculated for that loading direction divided by FL_FE calculated for the nominal axial loading direction (*θ* = 0°, *ϕ* = 0°), for each mouse at each time point.

To evaluate the effect of the time point (week 18 vs. week 20), and of the group (OVX vs. OVX + ML), the following quantities were calculated for all loading directions (*θ* in range 0°–30°, *ϕ* in range 0°–355°): percentage differences in failure load (ΔFLt_FE, %) between time points (week 18 vs. week 20) for OVX and OVX + ML groups, and the difference between the percentage changes in failure load (ΔFLg_FE, %) from week 18 to week 20 calculated between the two groups (OVX vs. OVX + ML).

A safety factor (SF) was calculated for each loading direction by dividing the calculated FL_FE by the applied load in the *in vivo* tibial loading model (12 N).

The apparent bone stiffness (S_FE, N/mm) was calculated as the ratio between the axial applied force (i.e., when *θ* = 0°) and the average displacement along the longitudinal direction calculated for the loaded nodes.

### 2.4 Statistical analysis

The mean, standard deviation (SD) and coefficient of variation (CV) of the apparent bone stiffness was calculated for the nominal axial loading direction for each group of mice, at each time point. The mean, SD and CV of the bone strengths were calculated for each loading direction for each group of mice, at each time point.

Non-parametric tests were chosen due to the results not being normally distributed (Shapiro-Wilks test) and the small sample size. The difference in stiffness between timepoints was assessed using the non-parametric two-tailed Wilcoxon test. The difference in stiffness between groups was assessed using the non-parametric two-tailed Mann-Whitney U test. The effect of the loading direction on the strength between time points was assessed using the non-parametric two-tailed Wilcoxon test. The effect of the loading direction on the strength between groups was assessed using the non-parametric two-tailed Mann-Whitney U test, as was the comparison between the nominal axial loading direction and every other loading direction. The statistical significance level was set at *α* = 0.05 for all tests.

## 3 Results

In total, 504 loading directions were evaluated for each mouse, for both mouse groups (OVX and OVX + ML) and for two time points (week 18 and week 20) (Table 1).

**TABLE 1:** For both groups and time points: loading directions associated with the minimum (*θ* = 30°, *ϕ* = 30°–50°) and maximum failure loads (*θ* = 10°, *ϕ* = 205°–210°), mean minimum and maximum failure loads (FL_FE), and mean apparent stiffness (S_FE) calculated for the nominal axial direction (*θ* = 0°, *ϕ* = 0°).

	Loading direction for minimum FL_FE (*θ*, range of *φ*) [°]	Minimum FL_FE (mean ± SD) [N]	Loading direction for maximum FL_FE (*θ*, range of *φ*) [°]	Maximum FL_FE (mean ± SD) [N]	S_FE (mean ± SD) [N/mm]
OVX-W18	30, [30–45]	12.6 ± 0.772	10, [205–210]	65.7 ± 4.78	243.2 ± 27.1
OVX-W20	30, [35–45]	12.5 ± 0.691	10, [205–210]	66.3 ± 4.40	237.0 ± 22.8
OVX + ML-W18	30, [40–45]	13.3 ± 0.606	10, [205–210]	69.5 ± 2.15	273.7 ± 8.0
OVX_ML-W20	30, [45–50]	15.1 ± 0.659	10, [205–210]	78.0 ± 3.34	293.0 ± 15.4

SD, standard deviation; OVX, ovariectomy; OVX + ML, ovariectomy and mechanical loading; W18, week 18; and W20, week 20.

The S_FE in the OVX group was not significantly different between week 18 (243.2 ± 27.1 N/mm) and week 20 (237.0 ± 22.8 N/mm; *p* = 0.188). The S_FE in the OVX + ML group at week 18 (273.7 ± 8.0 N/mm) was significantly different from the S_FE at week 20 (293.0 ± 15.4 N/mm, *p* = 0.031). The difference in the change of S_FE from week 18 to 20 was significantly higher for the OVX + ML (7.03% ± 2.66%) group compared to the OVX group (−2.55% ± 3.70%, *p* = 0.009).

**FIGURE 4:**
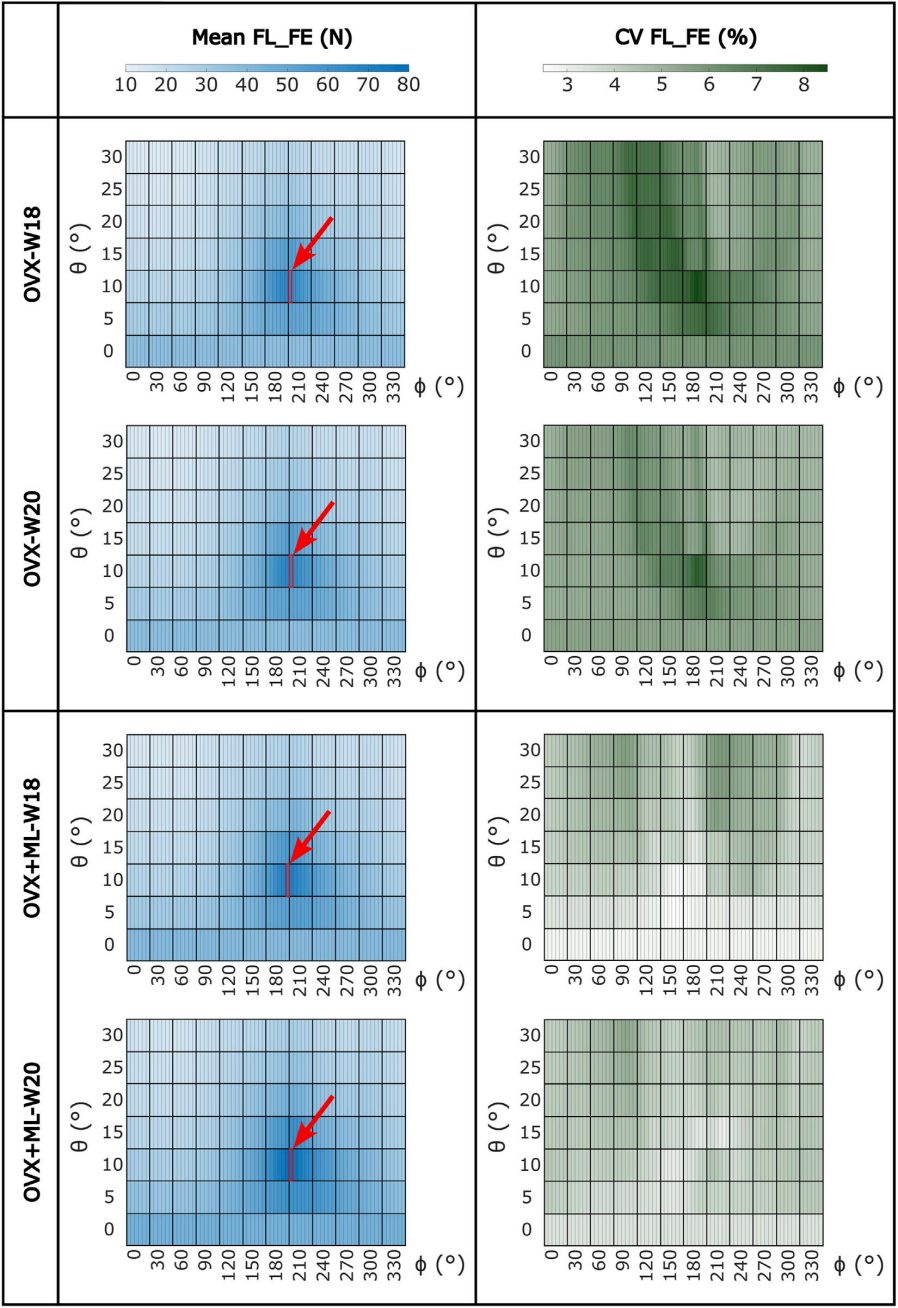
Heatmaps of the mean values of FL_FE and coefficients of variation (CV) across all loading directions (*θ* in range 0°–30°, *ϕ* in range 0°–355°) for both groups (OVX and OVX + ML) and time points (W18 and W20). The red square and arrow highlight the loading direction for which the maximum FL_FE was found. OVX, ovariectomy; OVX + ML, ovariectomy and mechanical loading; W18, week 18; W20, week 20.

Large variation in the FL_FE of the mouse tibiae across the tested loading directions was observed (Figure 4). Similar trends were found across all groups and time points. For the OVX group, at week 18, the FL_FE ranged from 12.6 N (*θ* = 30°, *ϕ* = 30°–45°) to 65.7 N (*θ* = 10°, *ϕ* = 205°–210°). At week 20, the FL_FE within the OVX group ranged from 12.5 N (*θ* = 30°, *ϕ* = 35°–45°) to 66.3 N (*θ* = 10°, *ϕ* = 205°–210°). For the OVX + ML group at week 18, the FL_FE ranged from 13.3 N (*θ* = 30°, *ϕ* = 40°–45°) to 69.5 N (*θ* = 10°, *ϕ* = 205°–210°). At week 20, the FL_FE in the OVX + ML group increased and ranged from 15.1 N (*θ* = 30°, *ϕ* = 45°–50°) to 78.0 N (similar angle for maximum FL_FE as for week 18; *θ* = 10°, *ϕ* = 205°–210°). The CV for the FL_FE for all groups and time points ranged between 2.49% and 8.54%, with the OVX group generally having larger coefficients of variation than the OVX + ML group.

**FIGURE 5:**
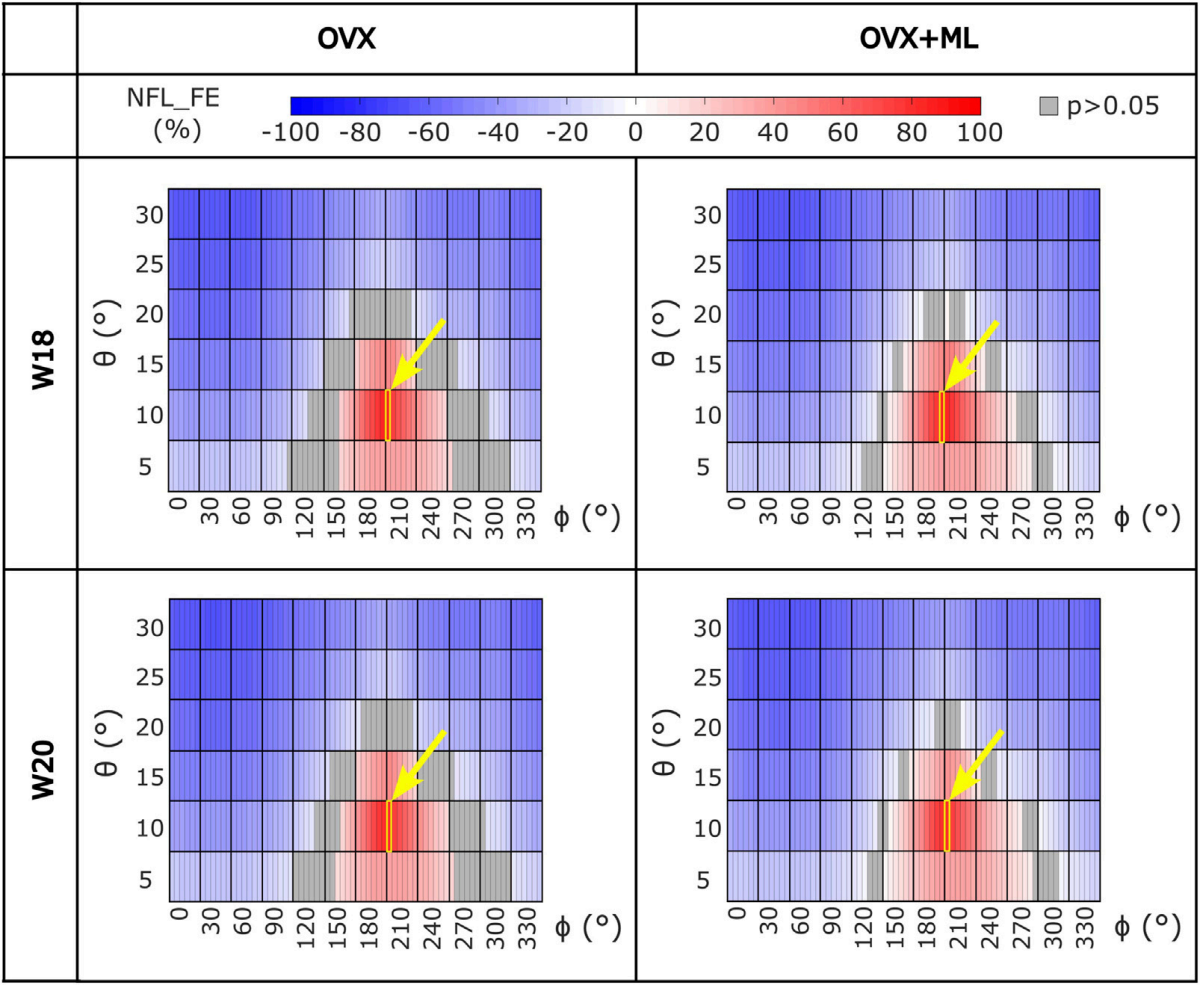
Heatmaps of the normalized failure load (NFL_FE) across all loading directions (*θ* in range 5°–30°, ϕ in range 0°–355°) for both groups (OVX and OVX + ML) and time points (W18 and W20). The values in grey show the loading directions associated with FL_FE to be not significantly different to the FL_FE obtained for the nominal axial loading direction. The yellow square and arrow highlight the loading direction for which the maximum NFL_FE was found. OVX, ovariectomy; OVX + ML, ovariectomy and mechanical loading; W18, week 18; W20, week 20.

Similar trends were found for the NFL_FE across all groups and time points (Figure 5). NFL_FE nearly doubled for loading directions with *θ* approximately 10° and *ϕ* between 205° and 210°.

**FIGURE 6:**
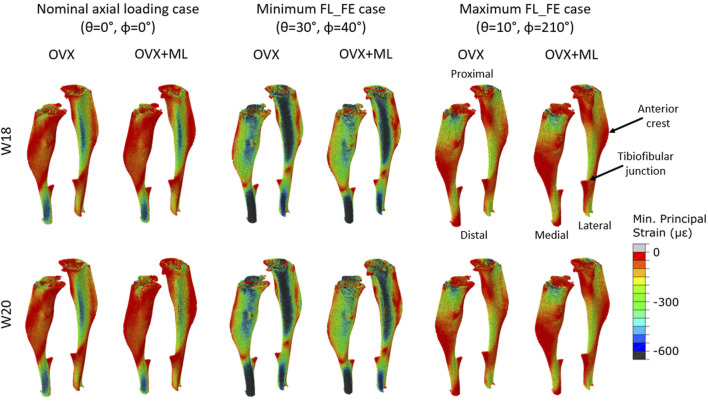
Minimum principal strain distribution of two representative tibiae (OVX–mouse 3, OVX + ML–mouse 5) obtained using a load of magnitude 1 N at W18 and W20. It should be noted that at W18 both groups were untreated, so observed differences are associated mainly with different animals. Left: Load applied along the nominal axial loading direction (*θ* = 0°, *ϕ* = 0°). Middle: Load applied along the loading direction associated with the minimum FL_FE (*θ* = 30°, *ϕ* = 40°). Right: Load applied along the loading direction associated with the maximum FL_FE (*θ* = 10°, *ϕ* = 210°). OVX, ovariectomy; OVX + ML, ovariectomy and mechanical loading; W18, week 18; W20–week 20.

**FIGURE 7:**
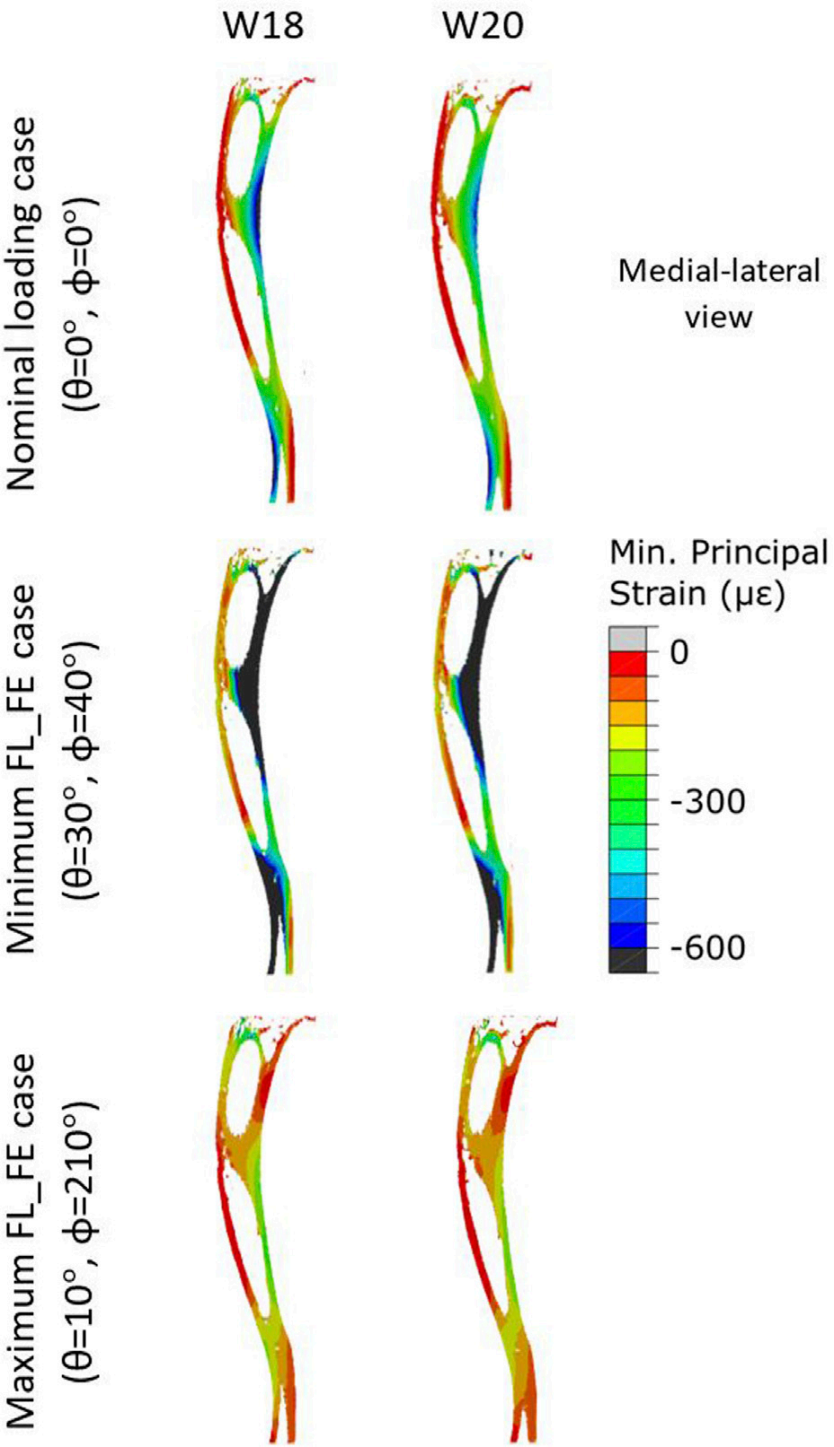
Internal minimum principal strain distribution of a representative tibia (mouse 5) within the OVX + ML group, obtained using a load magnitude 1 N at W18 and W20. For each week three loading directions are shown: the load applied along the nominal axial loading direction (*θ* = 0°, *ϕ* = 0°), the load applied along the loading direction associated with the minimum FL_FE (*θ* = 30°, *ϕ* = 40°), and the load applied along the loading direction associated with the maximum FL_FE (*θ* = 10°, *ϕ* = 210°). OVX, ovariectomy; W18, week 18; W20, week 20.

Typical distributions of the minimum principal strain obtained for loads along the nominal axial loading direction (*θ* = 0°, *ϕ* = 0°), and in the direction associated with the maximum FL_FE, are reported in Figure 6. For both the nominal axial loading direction and the loading direction associated with the minimum FL_FE and NFL_FE, high absolute values of the minimum principal strain were localized in the medial distal and lateral portions of the tibia. High strains were found proximally to the distal tibiofibular junction in the posterior portion of the bone, across all groups and time points. Lower strains were found at the anterior crest for all groups and time points. Additionally, for the loading direction associated with maximum FL_FE and NFL_FE, high absolute values of the minimum principal strain were also localized in the medial proximal portion of the tibia. As expected, small differences were found for models at week 18 between the two groups (both groups untreated), and larger differences induced by the mechanical loading could be observed at week 20. Typical internal distributions of the minimum principal strain calculated for models loaded along the nominal axial loading direction (*θ* = 0°, *ϕ* = 0°), along the direction associated with the minimum FL_FE, and along the direction associated with the maximum FL_FE, are reported in Figure 7. High absolute values of the minimum principal strain bridge the periosteal and endosteal surfaces of the cortical bone, for these three loading directions (a video showing all internal slices can be found in the Supplementary Material).

**FIGURE 8:**
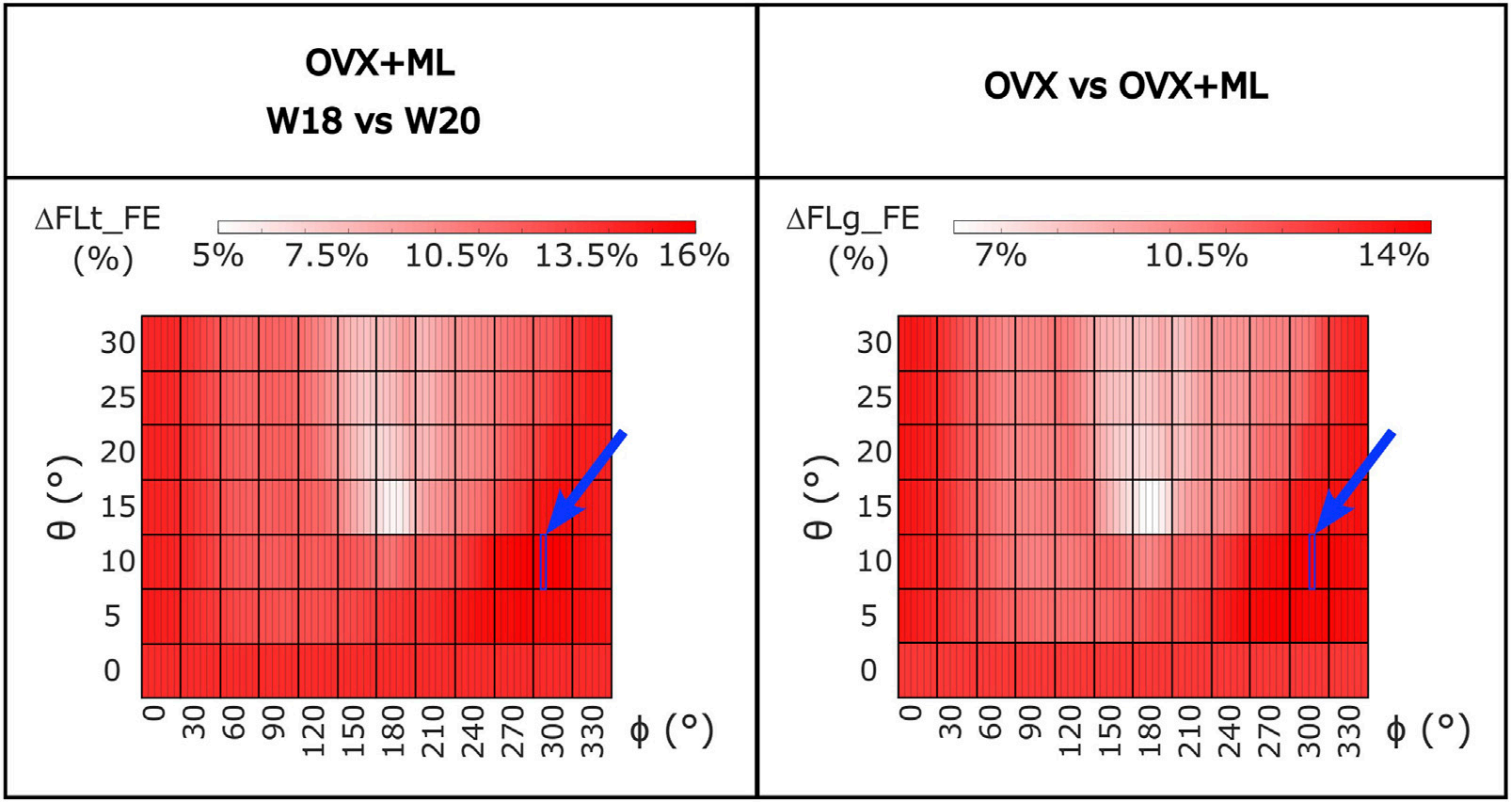
Left: Heatmap of the percentage difference in failure load (ΔFLt_FE) between time points (W18 vs. W20) for OVX + ML group for all loading directions (*θ* in range 0°–30°, *ϕ* in range 0°–355°). Right: Heatmap of the difference between the percentage difference in failure load (ΔFLg_FE) between W18 and W20 calculated between the two groups (OVX vs. OVX + ML) for all loading directions (*θ* in range 0°–30°, *ϕ* in range 0°–355°). All loading directions were statistically significant when the two time points were compared (Wilcoxon test, *p* < 0.05). The blue square and arrow highlight the loading direction for which the maximum difference was found. OVX, ovariectomy; OVX + ML, ovariectomy and mechanical loading; W18, week 18; W20, week 20.

For the OVX group, the FL_FE between weeks 18 and 20 (ΔFLt_FE) were not significantly different for any loading direction (maximum absolute difference 1.7%, *p* > 0.05; Supplementary Figure S1). For the OVX + ML group, the FL_FE increased significantly between weeks 18 and 20 (*p* < 0.031) with ΔFLt_FE between 5.4% (*θ* = 15°, *ϕ* = 190°) and 16.0% (*θ* = 10°, *ϕ* = 305°) (Figure 8). Percentage differences between ΔFLt_FE for the OVX and the OVX + ML groups were significant for every loading direction (*p* < 0.004), with ΔFLg_FE ranging between 6.2% (*θ* = 15°, *ϕ* = 190°) and 14.6% (*θ* = 10°, *ϕ* = 310°) (Figure 8).

The SF across all loading directions ranged from approximately 1 (in most cases for *θ* = 30° and *ϕ* between 35° and 50°) to 5–6 (*θ* = 10°, ϕ between 195° and 215°), in all groups and time points (Supplementary Figure S2).

## 4 Discussion

The overall goal of the study was to investigate the effect of the loading direction on the apparent mechanical properties of the mouse tibia and to evaluate how the apparent mechanical properties are sensitive to the load after the treatment with external mechanical loading.

Using validated micro-CT based micro-FE models, the apparent mechanical properties of the tibiae under different loading directions were calculated. The change over time of the apparent stiffness of the bone was higher for the OVX + ML group than the OVX group. A previous study, in which the experimental axial stiffness was measured from the load-displacement curves, showed the stiffness of C57BL/6 mice at ages 16 and 24 weeks to be in the range 188–307 N/mm for ovariectomized mice and in the range 234–366 N/mm for ovariectomized mice treated with PTH (Oliviero et al., 2021a). The high variability could be due to the heterogeneous effect of ovariectomy and PTH, but could also be due to experimental variables such as the bone alignment and fixation in the testing machine. Nevertheless, even though the age range of the mice in that study was larger (weeks 16–24) than that of this study (weeks 18–20) and the treatments vary between the two studies, the apparent stiffness values obtained in this study fit within the range of previously reported experimental stiffness values.

The values of the failure load for the different loading directions showed that the bone is stronger for a different loading direction to the nominal axial case (*θ* = 0°, *ϕ* = 0°), with the maximum occurring at *θ* equal to 10° and *ϕ* equal to 205° or 210°, suggesting that the bone is optimized for this loading direction. Considering that the fibula is located roughly at a *ϕ* angle between 170° and 220°, this result suggests that the fibula may share some of the load and reduce the deformation of the tibia if loaded along these directions. Nevertheless, it remains to be demonstrated to what extent the contribution of the fibula, proximal tibio-fibular joint, and soft tissues around the two bones, would affect the deformation of the tibia. The low coefficients of variation (range: 2.5%–8.5%) associated with the FL_FE estimations at each loading direction per time point, per group, highlight the reproducibility of the results, in line with the low errors associated with the generation of the micro-FE models, as recently reported by Oliviero et al. (2022). The mouse tibia has been shown to be very sensitive to the loading direction, with some loading directions resulting in a FL_FE ranging from half to double that of the nominal axial case. This result expands on those of a previous study, which showed that small variations of the load direction (effect of transverse load due to physiological loading on top of axial external mechanical loading) affect the local deformation and strain energy density (Cheong et al., 2021a). This result highlights the importance of controlling the loading direction when using the *in vivo* tibial loading model; a problem already demonstrated in a repositioning study for the *in vivo* tibial loading model (Giorgi and Dall’Ara, 2018) and in a misalignment study for the rodent tail loading model (Goff et al., 2014). The loading direction during this kind of experiment could be partially accounted for by integrating a tri-axial load cell in the experimental setup or using advanced 3D printed loading caps. It should be noted that the variability of the loading direction could be critical when developing multiscale models to predict bone adaptation driven by mechanoregulation (Pereira et al., 2015; Cheong et al., 2020b; Cheong et al., 2021b), and accounting for it may improve the accuracy of the predictions.

It should also be noted that the safety factor calculated in this study identified the loading directions with *θ* = 30° as critically close to 1, and therefore at risk of bone failure during the *in vivo* tibial loading experiments (Supplementary Figure S2). While further studies should be performed to understand the load sharing between the different musculoskeletal components of the mouse leg, this risk should be considered to maximize animal welfare during the experimental studies. Furthermore, the risks of inducing bone fractures during the tests would be reduced by incorporating features in the loading rigs that minimize potential misalignment errors, ensuring that they stay below 20° with respect to the longitudinal axis of the tibia.

When making comparisons between time points for the OVX group, the differences in FL_FE were within the limits of the inter-operator reproducibility error of the FE pipeline (precision error below 1.96%) (Oliviero et al., 2022), and in fact were not statistically different. This result confirms that the potential changes in bone geometry, trabecular bone density and partitional cortical bone density, and microstructure due to ovariectomy and growth between week 18 and week 20 do not affect the failure load, as previously reported by Roberts et al. (2019). However, this study extends the findings by confirming that this is valid for several loading directions. On the contrary, for the OVX + ML group, a statistically significant difference between the time points for all loading directions was found, with an increase of FL_FE between 5.4% and 16.0%. This was also confirmed by comparing longitudinally the OVX and OVX + ML groups, which highlighted differences in longitudinal changes of failure load between 6.2% and 14.6%. These results confirmed that the changes in morphometric and densitometric properties of the trabecular and cortical bone induced by the external mechanical loading (Roberts et al., 2020) translate into changes to the FL_FE for the different loading directions included in this study. Furthermore, the direction of the optimal load remains relatively constant (within 5°) across groups and time points (Figure 4). This result suggests that the mechanical loading increases the tibia FL_FE for all loading directions quite homogenously. However, when longitudinally comparing the two groups (OVX and OVX + ML), the largest difference was found for different loading directions (Figure 8), suggesting that the external mechanical loading may induce bone remodeling that leads to an improvement in FL_FE at non-optimized locations. Nevertheless, the orientation of the optimal load remains similar across time.

The main limitation of the study is that the model of the tibio-fibular complex has been simplified. Firstly, the fibula has not been included in the model. It is known that small differences in repositioning can lead to large transverse loads at the knee and ankle, which induce bending in the tibia (Giorgi and Dall’Ara, 2018). This is modelled by increasing *θ*, which results in an increase in the transverse loads, and this would in part be transferred through the fibula (Prasad et al., 2010; Cheong et al., 2021a). However, the inclusion of the fibula in the model would induce further assumptions, as the tibiofibular joint material properties are currently not known. Nevertheless, in typical *in vivo* tibial loading experiments, the bone remodeling associated with the fibula is also ignored and the aim of the experiment is to induce bone anabolism on the tibia by loading it axially. Additionally, the growth plate has not been included in the model. The main reason for this design choice is that currently little is known about the material properties of the growth plate. Nevertheless, this feature is likely to affect the transmission of the load in the proximal portion of the tibia, and may therefore affect the local deformation, and consequently the failure load, differently for the different loading directions.

In conclusion, this study has highlighted the importance of the loading direction on the failure load of the mouse tibia. The results of this study will be important to optimize the protocols for *in vivo* tibial loading experiments on mice. Moreover, external mechanical loading has been found to increase the bone strength across all loading directions, providing more insights on the effect of this intervention on the bone’s apparent mechanical properties.

## Data Availability

The raw data supporting the conclusion of this article will be made available by the authors, without undue reservation.

## References

[B1] Birkhold A. I. Razi H. Duda G. N. Weinkamer R. Checa S. Willie B. M. (2014). The influence of age on adaptive bone formation and bone resorption. Biomaterials 35, 9290–9301. 10.1016/J.BIOMATERIALS.2014.07.051 25128376

[B2] Bonewald L. F. (2011). The amazing osteocyte. J. Bone Mineral Res. 26, 229–238. 10.1002/JBMR.320 PMC3179345 21254230

[B3] Bouxsein M. L. Boyd S. K. Christiansen B. A. Guldberg R. E. Jepsen K. J. Müller R. (2010). Guidelines for assessment of bone microstructure in rodents using micro–computed tomography. J. Bone Mineral Res. 25, 1468–1486. 10.1002/JBMR.141 20533309

[B4] Bouxsein M. L. Myers K. S. Shultz K. L. Donahue L. R. Rosen C. J. Beamer W. G. (2005). Ovariectomy-induced bone loss varies among inbred strains of mice. J. Bone Mineral Res. 20, 1085–1092. 10.1359/JBMR.050307 15940361

[B5] Carriero A. Abela L. Pitsillides A. A. Shefelbine S. J. (2014). *Ex vivo* determination of bone tissue strains for an *in vivo* mouse tibial loading model. J. Biomech. 47, 2490–2497. 10.1016/J.JBIOMECH.2014.03.035 24835472 PMC4071445

[B6] Cheong V. S. Campos Marin A. Lacroix D. Dall’Ara E. (2020a). A novel algorithm to predict bone changes in the mouse tibia properties under physiological conditions. Biomech. Model. Mechanobiol. 19, 985–1001. 10.1007/s10237-019-01266-7 31786678 PMC7203598

[B7] Cheong V. S. Kadirkamanathan V. Dall’Ara E. (2021a). The role of the loading condition in predictions of bone adaptation in a mouse tibial loading model. Front. Bioeng. Biotechnol. 9, 676867. 10.3389/FBIOE.2021.676867 34178966 PMC8225949

[B8] Cheong V. S. Roberts B. C. Kadirkamanathan V. Dall’Ara E. (2020b). Bone remodelling in the mouse tibia is spatio-temporally modulated by oestrogen deficiency and external mechanical loading: a combined *in vivo*/*in silico* study. Acta Biomater. 116, 302–317. 10.1016/J.ACTBIO.2020.09.011 32911105

[B9] Cheong V. S. Roberts B. C. Kadirkamanathan V. Dall’Ara E. (2021b). Positive interactions of mechanical loading and PTH treatments on spatio-temporal bone remodelling. Acta Biomater. 136, 291–305. 10.1016/J.ACTBIO.2021.09.035 34563722

[B10] De Souza R. L. Matsuura M. Eckstein F. Rawlinson S. C. F. Lanyon L. E. Pitsillides A. A. (2005). Non-invasive axial loading of mouse tibiae increases cortical bone formation and modifies trabecular organization: a new model to study cortical and cancellous compartments in a single loaded element. Bone 37, 810–818. 10.1016/J.BONE.2005.07.022 16198164

[B11] Du J. Hartley C. Brooke-Wavell K. Paggiosi M. A. Walsh J. S. Li S. (2021). High-impact exercise stimulated localised adaptation of microarchitecture across distal tibia in postmenopausal women. Osteoporos. Int. 32, 907–919. 10.1007/s00198-020-05714-4 33196852

[B12] Frost H. M. (2003). Bone’s mechanostat: a 2003 update. Anat. Rec. A Discov. Mol. Cell. Evol. Biol. 275, 1081–1101. 10.1002/AR.A.10119 14613308

[B13] Giorgi M. Dall’Ara E. (2018). Variability in strain distribution in the mice tibia loading model: a preliminary study using digital volume correlation. Med. Eng. Phys. 62, 7–16. 10.1016/j.medengphy.2018.09.001 30243888

[B14] Goff M. G. Chang K. L. Litts E. N. Hernandez C. J. (2014). The effects of misalignment during *in vivo* loading of bone: techniques to detect the proximity of objects in three-dimensional models. J. Biomech. 47, 3156–3161. 10.1016/J.JBIOMECH.2014.06.016 25001204

[B15] Gould S. E. Junttila M. R. De Sauvage F. J. (2015). Translational value of mouse models in oncology drug development. Nat. Med. 21 (21), 431–439. 10.1038/nm.3853 25951530

[B16] Holguin N. Brodt M. D. Sanchez M. E. Kotiya A. A. Silva M. J. (2013). Adaptation of tibial structure and strength to axial compression depends on loading history in Both C57BL/6 and BALB/c mice. Calcif. Tissue Int. 93, 211–221. 10.1007/s00223-013-9744-4 23708853 PMC3748612

[B17] Levchuk A. Zwahlen A. Weigt C. Lambers F. M. Badilatti S. D. Schulte F. A. (2014). The Clinical Biomechanics Award 2012 - presented by the European Society of Biomechanics: large scale simulations of trabecular bone adaptation to loading and treatment. Clin. Biomech. (Bristol, Avon) 29, 355–362. 10.1016/J.CLINBIOMECH.2013.12.019 24467970

[B18] Lu Y. Boudiffa M. Dall’Ara E. Bellantuono I. Viceconti M. (2016). Development of a protocol to quantify local bone adaptation over space and time: quantification of reproducibility. J. Biomech. 49, 2095–2099. 10.1016/J.JBIOMECH.2016.05.022 27262181

[B19] Lu Y. Boudiffa M. Dall’Ara E. Liu Y. Bellantuono I. Viceconti M. (2017). Longitudinal effects of Parathyroid Hormone treatment on morphological, densitometric and mechanical properties of mouse tibia. J. Mech. Behav. Biomed. Mater 75, 244–251. 10.1016/J.JMBBM.2017.07.034 28756285

[B20] Lynch M. E. Main R. P. Xu Q. Schmicker T. L. Schaffler M. B. Wright T. M. (2011). Tibial compression is anabolic in the adult mouse skeleton despite reduced responsiveness with aging. Bone 49, 439–446. 10.1016/J.BONE.2011.05.017 21642027 PMC3235401

[B21] Main R. P. Shefelbine S. J. Meakin L. B. Silva M. J. van der Meulen M. C. H. Willie B. M. (2020). Murine axial compression tibial loading model to study bone mechanobiology: implementing the model and reporting results. J. Orthop. Research® 38, 233–252. 10.1002/JOR.24466 31508836 PMC9344861

[B22] Martelli S. Beck B. Saxby D. Lloyd D. Pivonka P. Taylor M. (2020). Modelling human locomotion to inform exercise prescription for osteoporosis. Curr. Osteoporos. Rep. 18, 301–311. 10.1007/s11914-020-00592-5 32335858 PMC7250953

[B23] Martin R. B. Burr D. B. Sharkey N. A. (1998). Skeletal tissue mechanics. Skelet. Tissue Mech. 10.1007/978-1-4757-2968-9

[B24] Meakin L. B. Todd H. Delisser P. J. Galea G. L. Moustafa A. Lanyon L. E. (2017). Parathyroid hormone’s enhancement of bones’ osteogenic response to loading is affected by ageing in a dose- and time-dependent manner. Bone 98, 59–67. 10.1016/J.BONE.2017.02.009 28249797 PMC5404907

[B25] Melville K. M. Kelly N. H. Khan S. A. Schimenti J. C. Ross F. P. Main R. P. (2014). Female mice lacking estrogen receptor-alpha in osteoblasts have compromised bone mass and strength. J. Bone Mineral Res. 29, 370–379. 10.1002/JBMR.2082 24038209

[B26] Miller C. J. Trichilo S. Pickering E. Martelli S. Delisser P. Meakin L. B. (2021). Cortical thickness adaptive response to mechanical loading depends on periosteal position and varies linearly with loading magnitude. Front. Bioeng. Biotechnol. 9, 671606. 10.3389/fbioe.2021.671606 34222215 PMC8249932

[B27] Moustafa A. Sugiyama T. Prasad J. Zaman G. Gross T. S. Lanyon L. E. (2012). Mechanical loading-related changes in osteocyte sclerostin expression in mice are more closely associated with the subsequent osteogenic response than the peak strains engendered. Osteoporos. Int. 23, 1225–1234. 10.1007/s00198-011-1656-4 21573880 PMC3304063

[B28] Nepal A. K. Essen H. W. van Jongh R. T. de Schoor N. M. van Otten R. H. J. Vanderschueren D. (2023). Methodological aspects of *in vivo* axial loading in rodents: a systematic review. J. Musculoskelet. Neuronal Interact. 23, 236–262. 37259664 PMC10233220

[B29] Oliviero S. Cheong V. S. Roberts B. C. Orozco Diaz C. A. Griffiths W. Bellantuono I. (2022). Reproducibility of densitometric and biomechanical assessment of the mouse tibia from *in vivo* micro-CT images. Front. Endocrinol. (Lausanne) 13, 915938. 10.3389/fendo.2022.915938 35846342 PMC9282377

[B30] Oliviero S. Giorgi M. Dall’Ara E. (2018). Validation of finite element models of the mouse tibia using digital volume correlation. J. Mech. Behav. Biomed. Mater 86, 172–184. 10.1016/J.JMBBM.2018.06.022 29986291

[B31] Oliviero S. Giorgi M. Laud P. J. Dall’Ara E. (2019). Effect of repeated *in vivo* microCT imaging on the properties of the mouse tibia. PLoS One 14, e0225127. 10.1371/JOURNAL.PONE.0225127 31751367 PMC6874075

[B32] Oliviero S. Lu Y. Viceconti M. Dall’Ara E. (2017). Effect of integration time on the morphometric, densitometric and mechanical properties of the mouse tibia. J. Biomech. 65, 203–211. 10.1016/j.jbiomech.2017.10.026 29126603

[B33] Oliviero S. Owen R. Reilly G. C. Bellantuono I. Dall’Ara E. (2021a). Optimization of the failure criterion in micro-Finite Element models of the mouse tibia for the non-invasive prediction of its failure load in preclinical applications. J. Mech. Behav. Biomed. Mater 113, 104190. 10.1016/J.JMBBM.2020.104190 33191174

[B34] Oliviero S. Roberts M. Owen R. Reilly G. C. Bellantuono I. Dall’Ara E. (2021b). Non-invasive prediction of the mouse tibia mechanical properties from microCT images: comparison between different finite element models. Biomech. Model. Mechanobiol. 20, 941–955. 10.1007/S10237-021-01422-Y 33523337 PMC8154847

[B35] O’Rourke D. Beck B. R. Harding A. T. Watson S. L. Pivonka P. Martelli S. (2021). Assessment of femoral neck strength and bone mineral density changes following exercise using 3D-DXA images. J. Biomech. 119, 110315. 10.1016/J.JBIOMECH.2021.110315 33636460

[B36] Patel T. K. Brodt M. D. Silva M. J. (2014). Experimental and finite element analysis of strains induced by axial tibial compression in young-adult and old female C57Bl/6 mice. J. Biomech. 47, 451–457. 10.1016/J.JBIOMECH.2013.10.052 24268312 PMC3902696

[B37] Pereira A. F. Javaheri B. Pitsillides A. A. Shefelbine S. J. (2015). Predicting cortical bone adaptation to axial loading in the mouse tibia. J. R. Soc. Interface 12, 20150590. 10.1098/RSIF.2015.0590 26311315 PMC4614470

[B38] Pickering E. Silva M. J. Delisser P. Brodt M. D. Gu Y. T. Pivonka P. (2021). Estimation of load conditions and strain distribution for *in vivo* murine tibia compression loading using experimentally informed finite element models. J. Biomech. 115, 110140. 10.1016/J.JBIOMECH.2020.110140 33348259 PMC7856106

[B39] Prasad J. Wiater B. P. Nork S. E. Bain S. D. Gross T. S. (2010). Characterizing gait induced normal strains in a murine tibia cortical bone defect model. J. Biomech. 43, 2765–2770. 10.1016/J.JBIOMECH.2010.06.030 20674920

[B40] Razi H. Birkhold A. I. Weinkamer R. Duda G. N. Willie B. M. Checa S. (2015a). Aging leads to a dysregulation in mechanically driven bone formation and resorption. J. Bone Mineral Res. 30, 1864–1873. 10.1002/JBMR.2528 25857303

[B41] Razi H. Birkhold A. I. Zaslansky P. Weinkamer R. Duda G. N. Willie B. M. (2015b). Skeletal maturity leads to a reduction in the strain magnitudes induced within the bone: a murine tibia study. Acta Biomater. 13, 301–310. 10.1016/J.ACTBIO.2014.11.021 25463494

[B42] Roberts B. C. Carrera H. M. A. Zanjani-Pour S. Boudiffa M. Wang N. Gartland A. (2020). PTH(1-34) treatment and/or mechanical loading have different osteogenic effects on the trabecular and cortical bone in the ovariectomized C57BL/6 mouse. Sci. Rep. 10, 8889. 10.1038/s41598-020-65921-1 32483372 PMC7264307

[B43] Roberts B. C. Cheong V. S. Oliviero S. Carrera H. M. A. Wang N. Gartland A. (2023). Combining PTH(1-34) and mechanical loading has increased benefit to tibia bone mechanics in ovariectomised mice. J. Orthop. Research®. 10.1002/JOR.25777 38151816

[B44] Roberts B. C. Giorgi M. Oliviero S. Wang N. Boudiffa M. Dall’Ara E. (2019). The longitudinal effects of ovariectomy on the morphometric, densitometric and mechanical properties in the murine tibia: a comparison between two mouse strains. Bone 127, 260–270. 10.1016/J.BONE.2019.06.024 31254730

[B45] Rooney A. M. McNeill T. J. Ross F. P. Bostrom M. P. G. van der Meulen M. C. H. (2023). PTH treatment increases cortical bone mass more in response to compression than tension in mice. J. Bone Mineral Res. 38, 59–69. 10.1002/JBMR.4728 PMC12054691 36281491

[B46] Scheuren A. C. Vallaster P. Kuhn G. A. Paul G. R. Malhotra A. Kameo Y. (2020). Mechano-regulation of trabecular bone adaptation is controlled by the local *in vivo* environment and logarithmically dependent on loading frequency. Front. Bioeng. Biotechnol. 8, 566346. 10.3389/FBIOE.2020.566346 33154964 PMC7591723

[B47] Shane E. Burr D. Abrahamsen B. Adler R. A. Brown T. D. Cheung A. M. (2014). Atypical subtrochanteric and diaphyseal femoral fractures: second report of a task force of the American society for bone and mineral research. J. Bone Mineral Res. 29, 1–23. 10.1002/JBMR.1998 23712442

[B48] Stadelmann V. Hocke J. Verhelle J. Forster V. Merlini F. Terrier A. (2009). Walk-to-run transition: about the Modela dimensionless number. Comput. Methods Biomech. Biomed. Engin 12, 95–96. 10.1080/10255840903077287 18651261

[B49] Sugiyama T. Meakin L. B. Browne W. J. Galea G. L. Price J. S. Lanyon L. E. (2012). Bones’ adaptive response to mechanical loading is essentially linear between the low strains associated with disuse and the high strains associated with the lamellar/woven bone transition. J. Bone Mineral Res. 27, 1784–1793. 10.1002/JBMR.1599 PMC3427886 22431329

[B50] Sugiyama T. Price J. S. Lanyon L. E. (2010). Functional adaptation to mechanical loading in both cortical and cancellous bone is controlled locally and is confined to the loaded bones. Bone 46, 314–321. 10.1016/J.BONE.2009.08.054 19733269 PMC2825292

[B51] Sugiyama T. Saxon L. K. Zaman G. Moustafa A. Sunters A. Price J. S. (2008). Mechanical loading enhances the anabolic effects of intermittent parathyroid hormone (1–34) on trabecular and cortical bone in mice. Bone 43, 238–248. 10.1016/J.BONE.2008.04.012 18539556

[B52] Viceconti M. Dall’Ara E. (2019). From bed to bench: how *in silico* medicine can help ageing research. Mech. Ageing Dev. 177, 103–108. 10.1016/J.MAD.2018.07.001 30005915

[B53] Yang H. Butz K. D. Duffy D. Niebur G. L. Nauman E. A. Main R. P. (2014). Characterization of cancellous and cortical bone strain in the *in vivo* mouse tibial loading model using microCT-based finite element analysis. Bone 66, 131–139. 10.1016/J.BONE.2014.05.019 24925445

[B54] Yang H. Embry R. E. Main R. P. (2017). Effects of loading duration and short rest insertion on cancellous and cortical bone adaptation in the mouse tibia. PLoS One 12, e0169519. 10.1371/JOURNAL.PONE.0169519 28076363 PMC5226737

[B55] Yeam C. T. Chia S. Tan H. C. C. Kwan Y. H. Fong W. Seng J. J. B. (2018). A systematic review of factors affecting medication adherence among patients with osteoporosis. Osteoporos. Int. 29 (29), 2623–2637. 10.1007/S00198-018-4759-3 30417253

