# Supplementary material for: The loading direction dramatically affects the mechanical properties of the mouse tibia
Source: Front Bioeng Biotechnol. 2024 Feb 6;12:1335955. doi: 10.3389/fbioe.2024.1335955 (PMC10877372; doi:10.3389/fbioe.2024.1335955)
Supplement: Supplementary file 1 [file DataSheet1.pdf]

Supplementary Material

1.1 Supplementary Figure 1

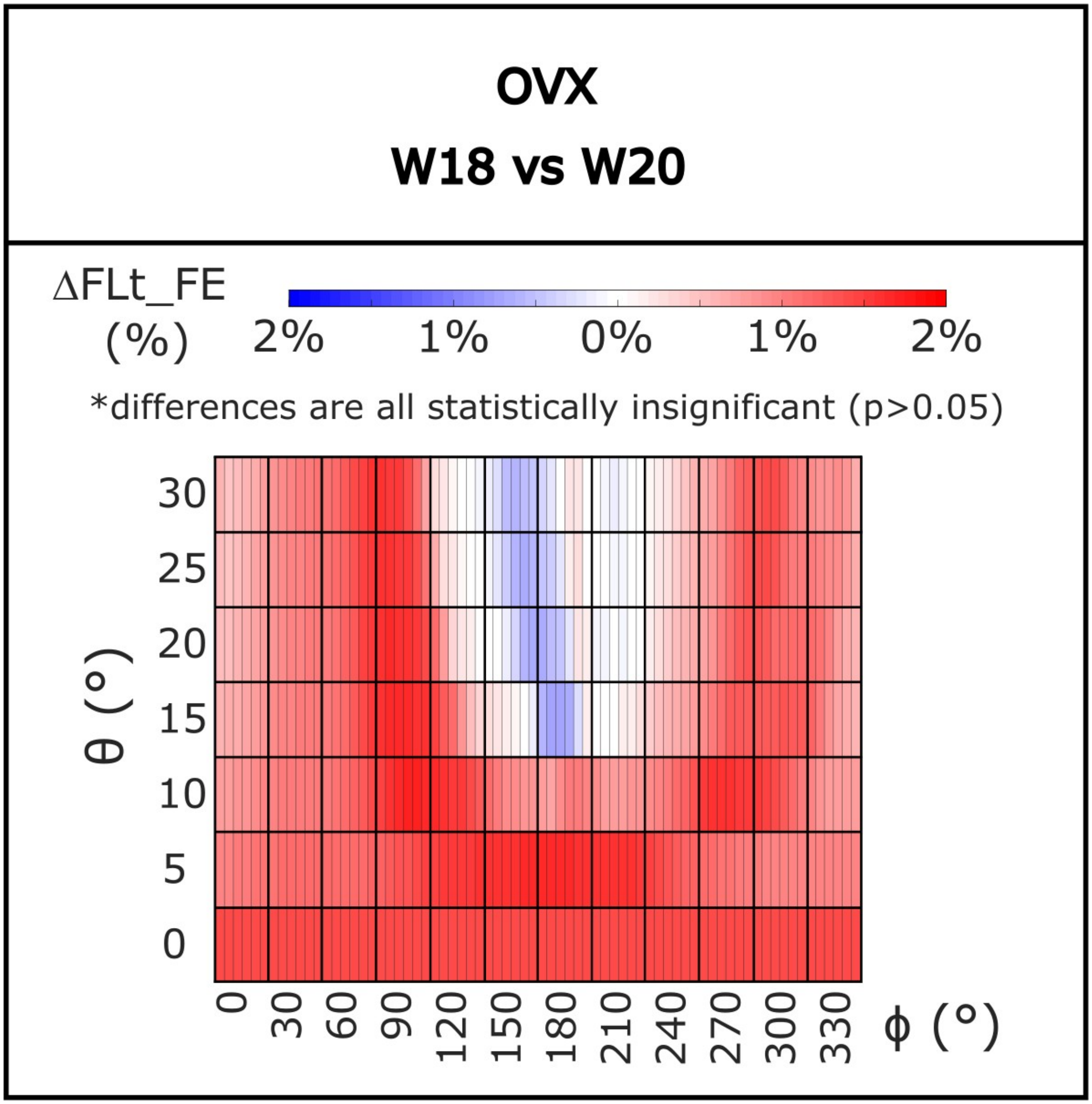

Supplementary Figure 1: Heatmap of the percentage difference in failure load (ΔFLt_FE) between timepoints (W18 vs W20) for OVX group for all loading directions (θ in range 0-30°, ϕ in range 0-355°). Differences are all statistically insignificant (Wilcoxon test, $p < 0.05$). OVX – ovariectomy, W18 – week 18, W20 – week 20.

1.2 Supplementary Figure 2

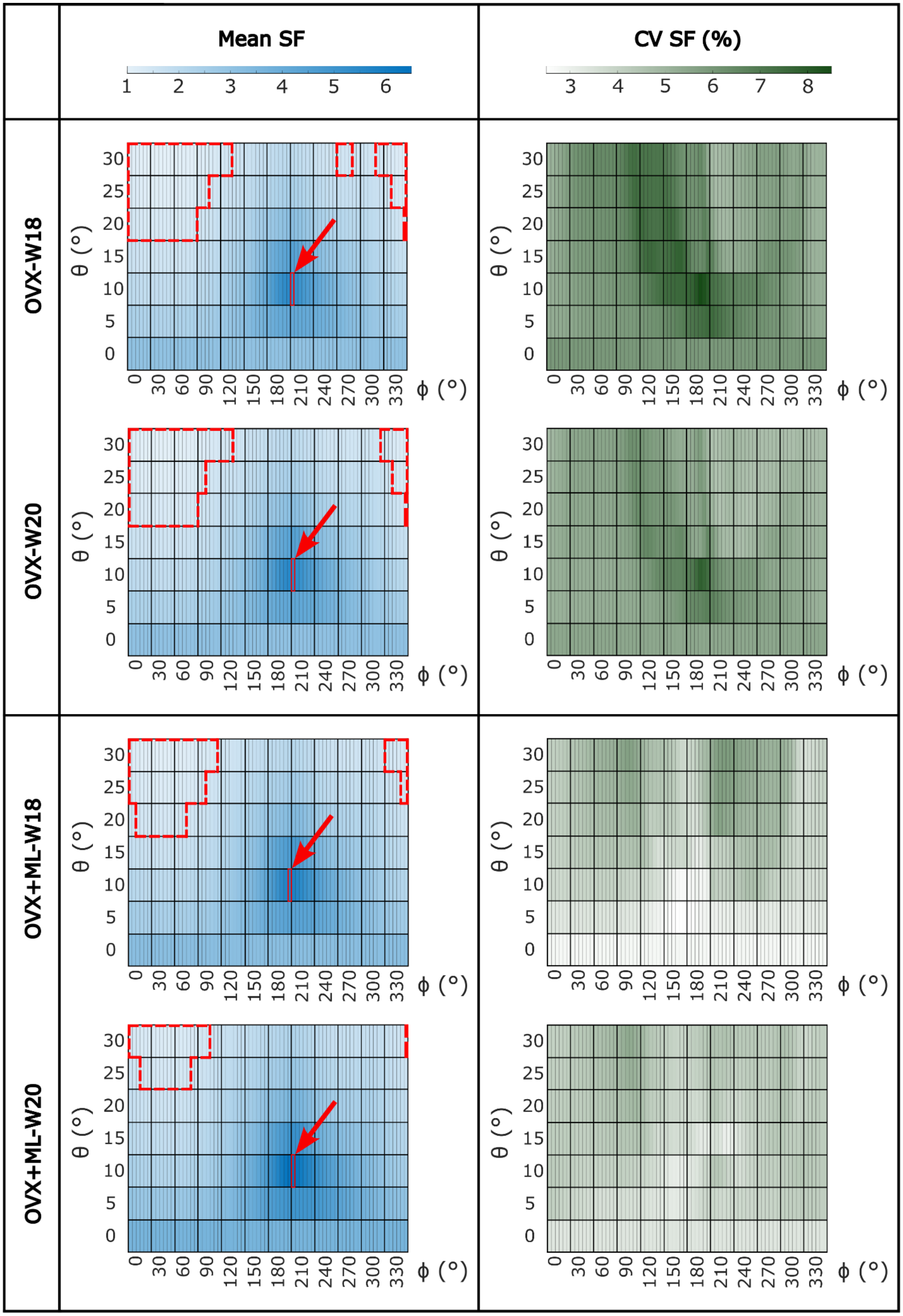
Mean SF
1 2 3 4 5 6
CV SF (%)
3 4 5 6 7 8
OVX-W18
OVX-W20
OVX+ML-W18
OVX+ML-W20
θ (°)
30 25 20 15 10 5 0
ϕ (°)
0 30 60 90 120 150 180 210 240 270 300 330

Supplementary Figure 2: Heatmaps of the mean values of safety factor (SF) and coefficients of variation (CV, %) across all loading directions (θ = 0-30°, ϕ = 0-355°) for both groups and timepoints. The red square and arrow highlight the loading direction for which the maximum safety factor was found, and the dashed red areas highlight the loading directions where the safety factor is between 1 and 1.5. OVX – ovariectomy, OVX+ML – ovariectomy and mechanical loading, W18 – week 18, and W20 – week 20.

1.3 Supplementary Video 1

Please see the video in the Supplementary Material.

Supplementary Video 1: Internal minimum principal strain distribution of a representative tibia (mouse 5) within the OVX+ML group, obtained using a load with magnitude 1 N at W18 and W20. The video shows the 2D strain distributions slice by slice, from the lateral to the medial side of the tibiae. For each week two loading directions are shown: the load applied along the nominal axial loading direction (θ = 0°, ϕ = 0°) and the load applied along the loading direction associated with the maximum FL_FE (θ = 10°, ϕ = 210°). OVX – ovariectomy, OVX+ML – ovariectomy and mechanical loading, W18 – week 18, W20 – week 20.

frontiers
